# Supplementary material for: Dynamic stroma reorganization drives blood vessel dysmorphia during glioma growth
Source: EMBO Mol Med. 2017 Oct 16;9(12):1629–45. doi: 10.15252/emmm.201607445 (PMC5709745; doi:10.15252/emmm.201607445)
Supplement: Supplementary file 1 — Appendix [file EMMM-9-1629-s001.pdf]

Appendix

Authors:

Thomas Mathivet[1,2,12]; Claire Bouleti[1,2]; Matthias Van Woensel[3]; Fabio Stanchi[1,2]; Tina Verschuere[3]; Li-Kun Phng[1,2,4]; Joost Dejaegher[3]; Marly Balcer[1,2]; Ken Matsumoto[1,2]; Petya Georgieva[1,2]; Jochen Belmans[3]; Raf Sciot[5]; Christian Stockmann[6], Massimiliano Mazzone[7,8]; Steven De Vleeschouwer[3,9]; Holger Gerhardt[1,2,10,11,12].

Affiliations:

[1] Vascular Patterning Lab, Center for Cancer Biology, VIB, Leuven, Belgium

[2] Vascular Patterning Lab, Department of Oncology, KU Leuven, Leuven, Belgium

[3] Department of Neurosciences, Laboratory of Experimental Neurosurgery and Neuroanatomy, KU Leuven, Leuven, Belgium

[4] Laboratory for Vascular Morphogenesis, RIKEN Center for Developmental Biology, Kobe, Japan.

[5] Department of Pathology, KU Leuven and University Hospitals Leuven, Leuven, Belgium.

[6] UMR 970, Paris Cardiovascular Research Center, Institut National de la Santé et de la Recherche Médicale (INSERM), Paris, France

[7] Lab of Molecular Oncology and Angiogenesis, Department of Oncology, KU Leuven, Leuven, Belgium

[8] Lab of Molecular Oncology and Angiogenesis, Center for Cancer Biology, VIB, Leuven, Belgium

[9] Department of Neurosciences, KU Leuven, Leuven, Belgium

[10] Integrative Vascular Biology Laboratory, Max-Delbrück-Center for Molecular Medicine in the Helmholtz Association (MDC), 13125 Berlin, Germany

[11] Berlin Institute of Health (BIH), Berlin, Germany.

[12] Corresponding authors.

Table of content:

Appendix figures:

Appendix Figure S1 linked to Figure 2 *(Immune cells recruitment in glioma).*

Appendix Figure S2 linked to Figure 2 *(LifeAct-GFP bone marrow transplant and CSF1R-Mer-Cre-Mer-GFP labeled macrophages are specific from bone marrow derived macrophages and do not label resident macrophages).*

Appendix Figure S3 linked to Figure 2 *(Bone marrow derived macrophage proliferation in the glioma microenvironment).*

Appendix Figure S4 linked to Figure 2 *(Iba1 positive resident macrophages mainly stay at the glioma margin compared to tumor core invading bone marrow derived F4/80 positive macrophages).*

Appendix Figure S5 linked to Figure 3 *(MHCII and MRC1 are segregating markers of M1 and M2 macrophages respectively).*

Appendix Figure S6 linked to Figure 4 *(Blood vessel leakage in late stage glioma in human).*

Appendix Figure S7 linked to Figure 5 *(Concomitant neutrophils and myeloid-derived suppressor cells (MDSCs) recruitment with macrophages recruitment inhibition).*

Appendix Figure S8 linked to Figure 7 *(Hypoxic area increases during glioma growth).*

Appendix Figure S9 independent *(anti-CSF1 treatment reduce vessel caliber and slightly increase tumor growth in melanoma model).*

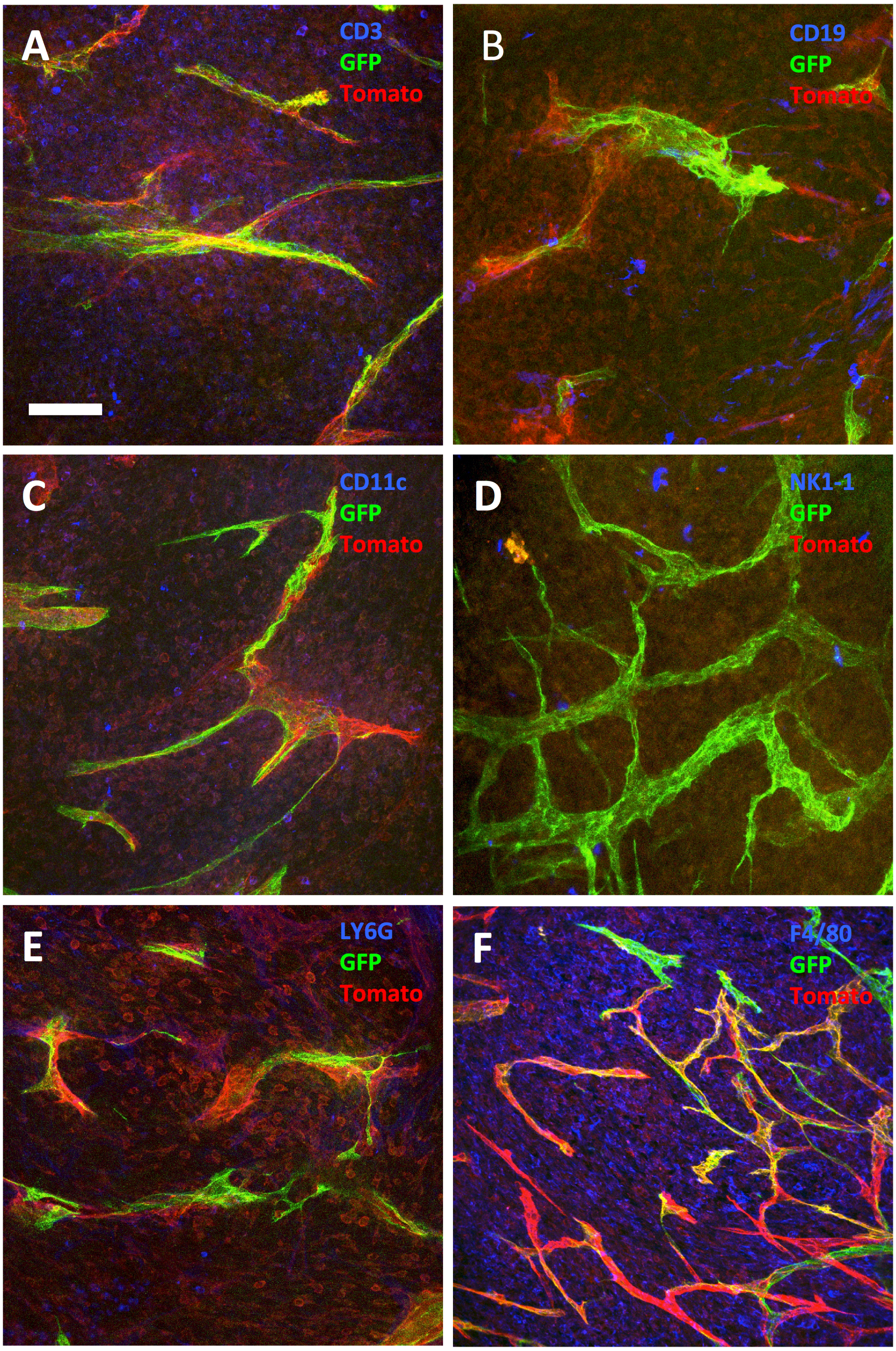

Appendix Figure S1: Immune cells recruitment in glioma. CD3 (**A**), CD19 (**B**), CD11c (**C**), NK1-1 (**D**), LY6G (**E**) and F4/80 (**F**) immunohistochemistry on 5 weeks growth glioma section implanted in ROSA^{mTmG}::*Pdgfb-iCre* mouse respectively staining T-cells, B-cells, dendritic cells, natural killer cells, neutrophils and macrophages (50µm depth stack). Scale bar: 80µm.

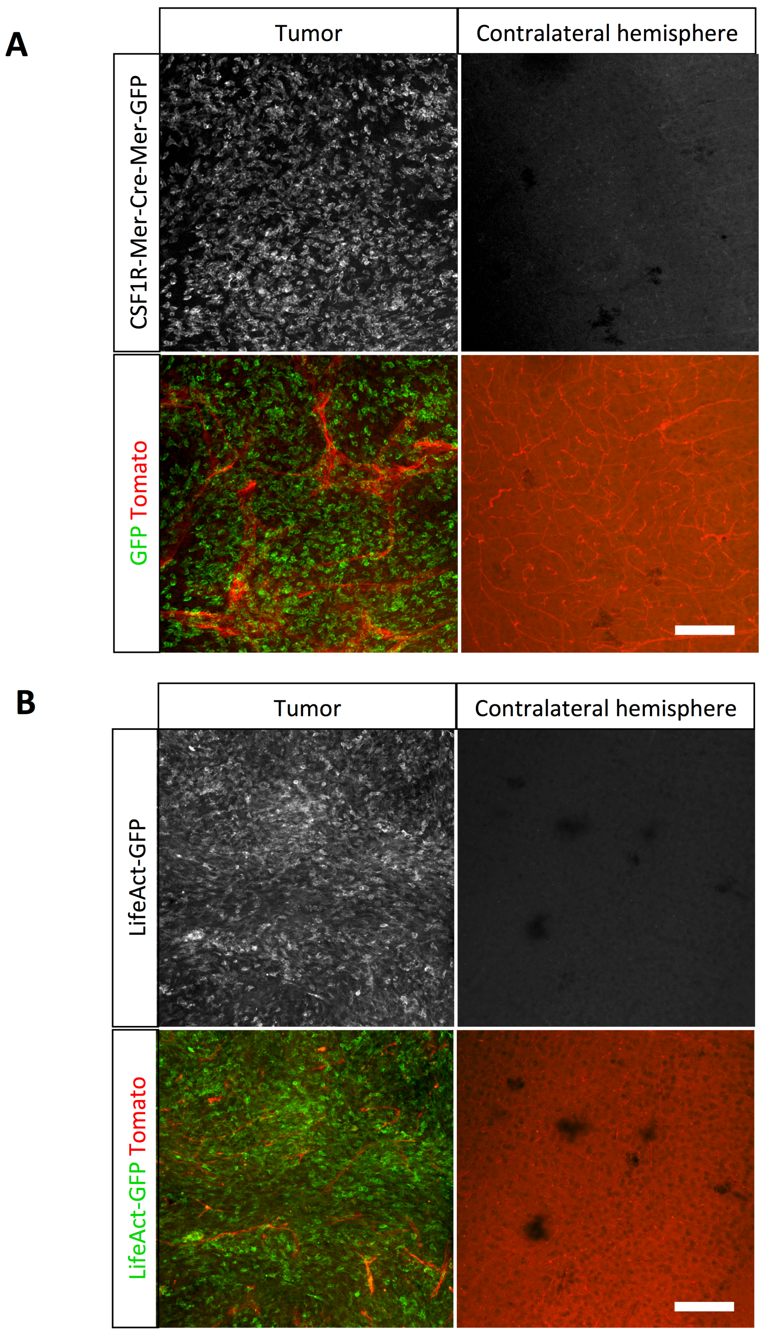

Appendix Figure S2: LifeAct-GFP bone marrow transplant and CSF1R-Mer-Cre-Mer-GFP labeled macrophages are specific from bone marrow derived macrophages and do not label resident macrophages. A. Confocal imaging of LifeAct-GFP bone marrow transplantation in the tumor and the contralateral hemisphere of 5 weeks implanted glioma in ROSA$^{mT/mG}$ mice. **B.** Confocal imaging of the tumor and the contralateral hemisphere of 5 weeks implanted glioma in ROSA^{mTmG}*::Csf1r-Mer-iCre-Mer* mouse. Scale bar: 150µm.

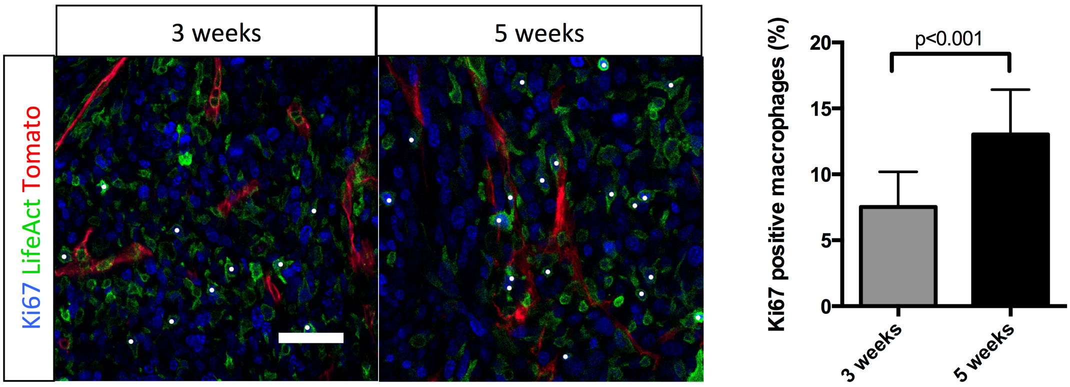

Appendix Figure S3: Bone marrow derived macrophage proliferation in the glioma microenvironment. Ki67 immunohistochemistry on 3 and 5 weeks growth glioma of LifeAct-GFP bone marrow transplantation in ROSA$^{mT/mG}$ mice (n=4 mice per group) (50µm depth stack). Statistical analysis: t-test. Error bars: mean±SD. Scale bars: 100µm.

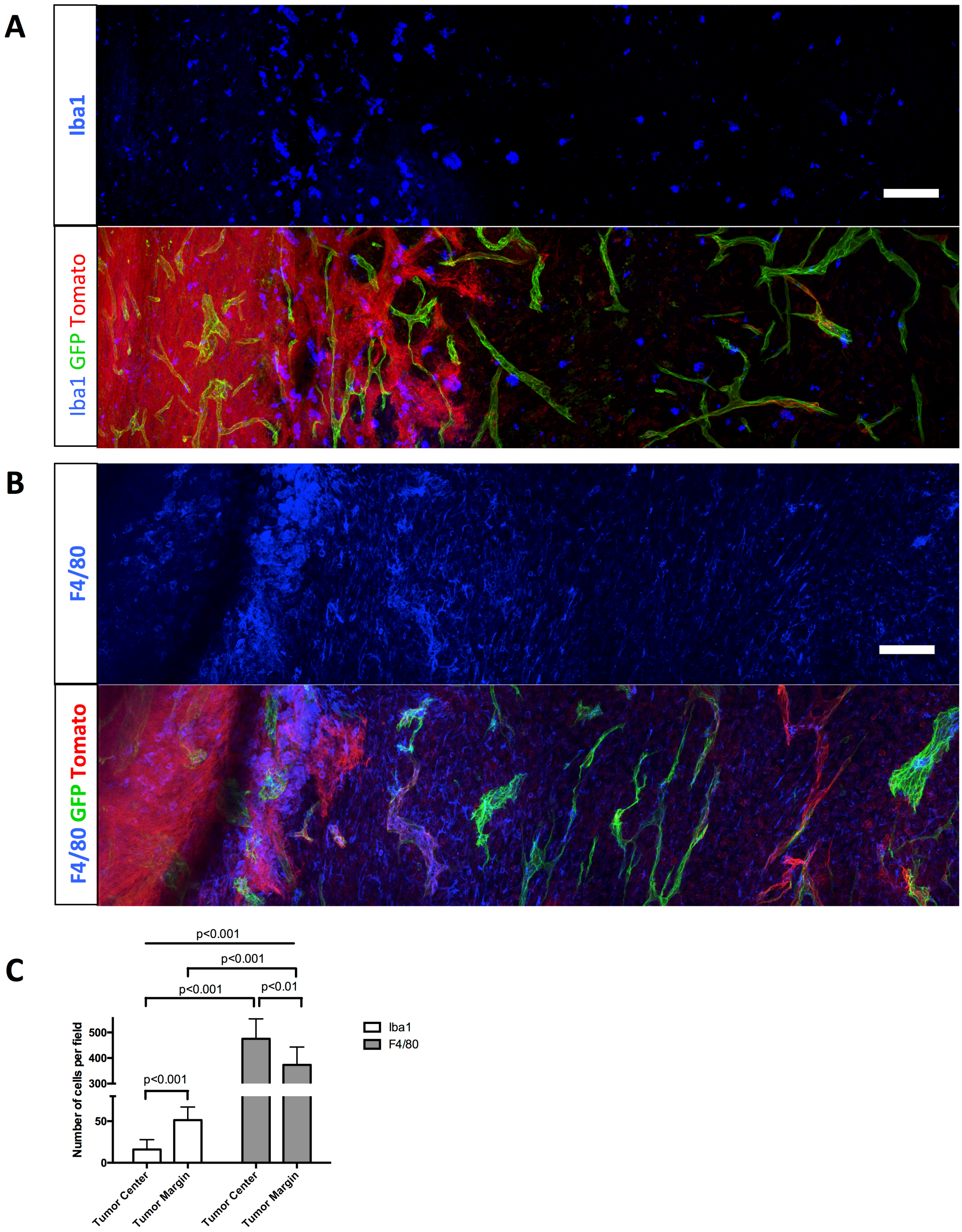

Appendix Figure S4: Iba1 positive resident macrophages mainly stay at the glioma margin compared to tumor core invading bone marrow derived F4/80 positive macrophages. Iba1 (A) and F4/80 (B) immunohistochemistry 5 weeks growth glioma in ROSA^{mTmG}::*Pdgfb-iCre* mouse (50µm depth stack). C. Quantification of Iba1 or F4/80 positive cells in tumor center or tumor margin areas. Statistical analysis: C. two-way ANOVA followed by multiple comparisons Tukey's test. Error bars: mean±SD. Scale bar: 250µm.

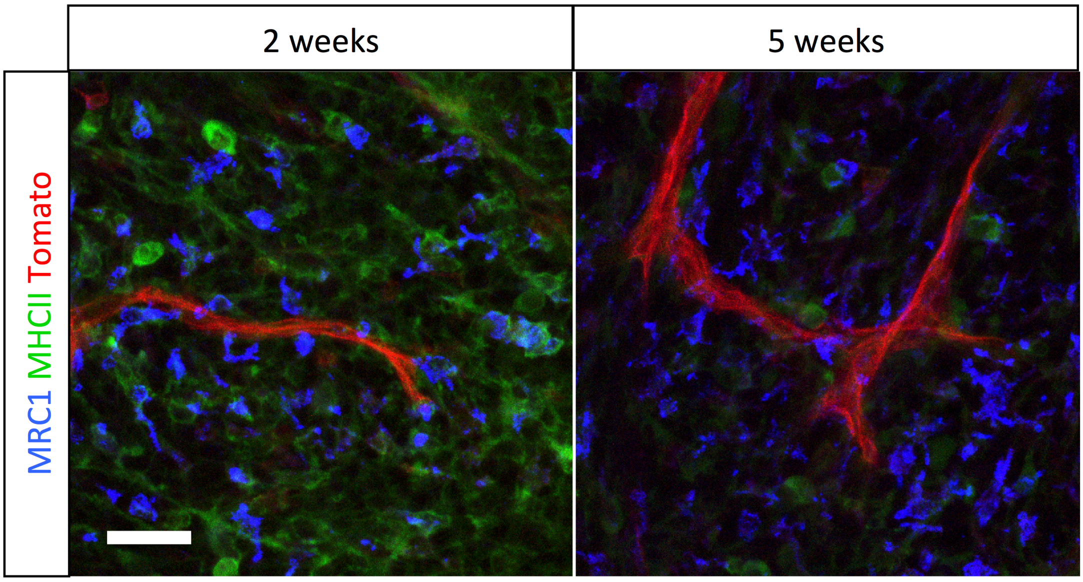

Appendix Figure S5: MHCII and MRC1 are segregating markers of M1 and M2 macrophages respectively. MHCII and MRC1 immunohistochemistry on 2 and 5 weeks growth glioma in ROSA^{mTmG}*::Pdgfb-iCre* mouse (20µm depth stack). Scale bar: 80µm.

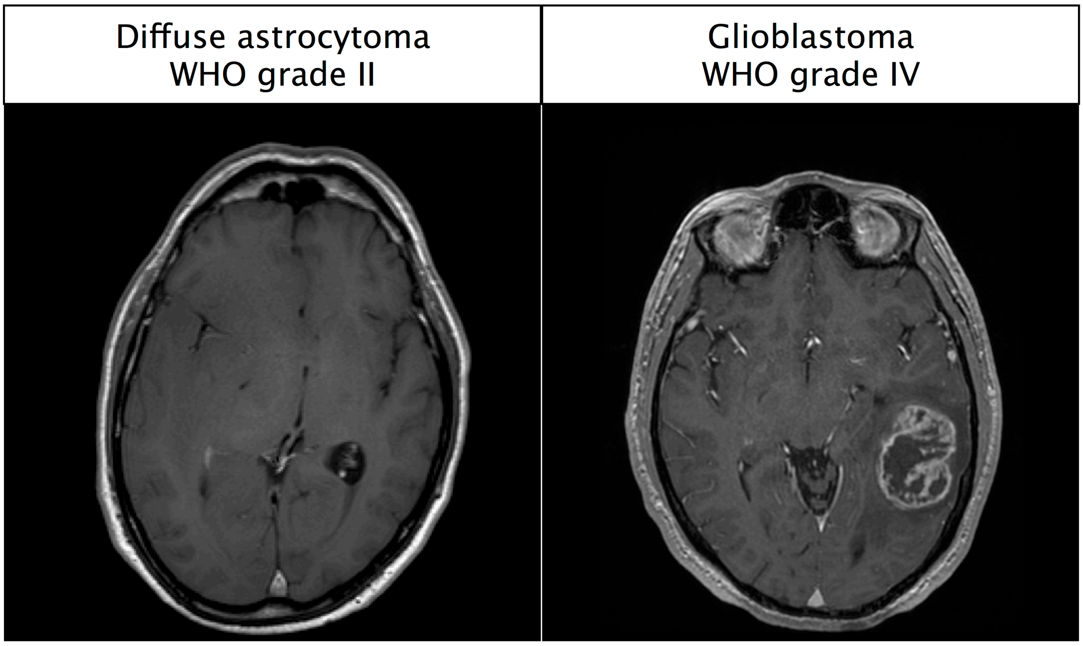

Appendix Figure S6: Blood vessel leakage in late stage glioma in human. MRI imaging in gadolinium administered patient bearing grade II and grade IV glioma. Gadolinium extravasates from the blood stream in glioblastoma (grade IV) but not in glioma grade II.

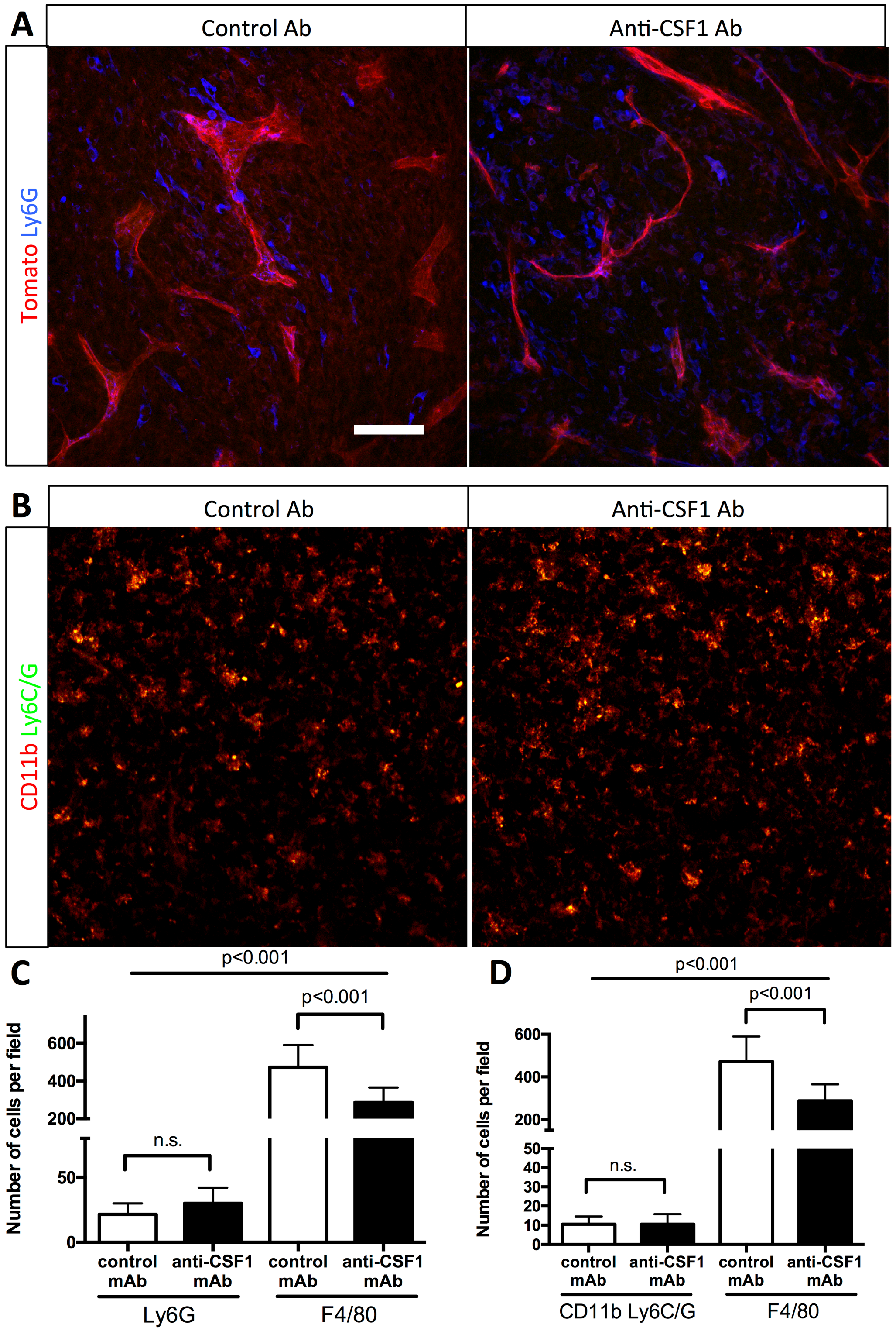

Figure S7: Concomitant neutrophils and myeloid-derived suppressor cells (MDSCs) recruitment with macrophages recruitment inhibition. A. Ly6G immunohistochemistry on sections of 4 weeks growth glioma in ROSA$^{mT/mG}$ mice treated with anti-CSF1 or control antibodies (50µm depth stack). **B.** CD11b Ly6C/G immunohistochemistry on sections of 4 weeks

growth glioma treated with anti-CSF1 or control antibodies (50µm depth stack). **C.** Quantification of Ly6G positive cells in response to anti-CSF1 mAb treatment in comparison to F4/80 positive cells. Anti-CSF1 antibody treatment induces a tendential 1.4 fold increase in neutrophil recruitment compared to control antibody (n=6 mice per group) **D.** Quantification of CD11b Ly6C/G positive cells in response to anti-CSF1 treatment in comparison to F4/80 positive cells. Anti-CSF1 antibody treatment did not affect MDSCs recruitment compared to control antibody (n=6 mice per group). Statistical analysis: C.D. one-way ANOVA followed by multiple comparisons Tukey's test. Error bars: mean±SD. Scale bar: 70µm.

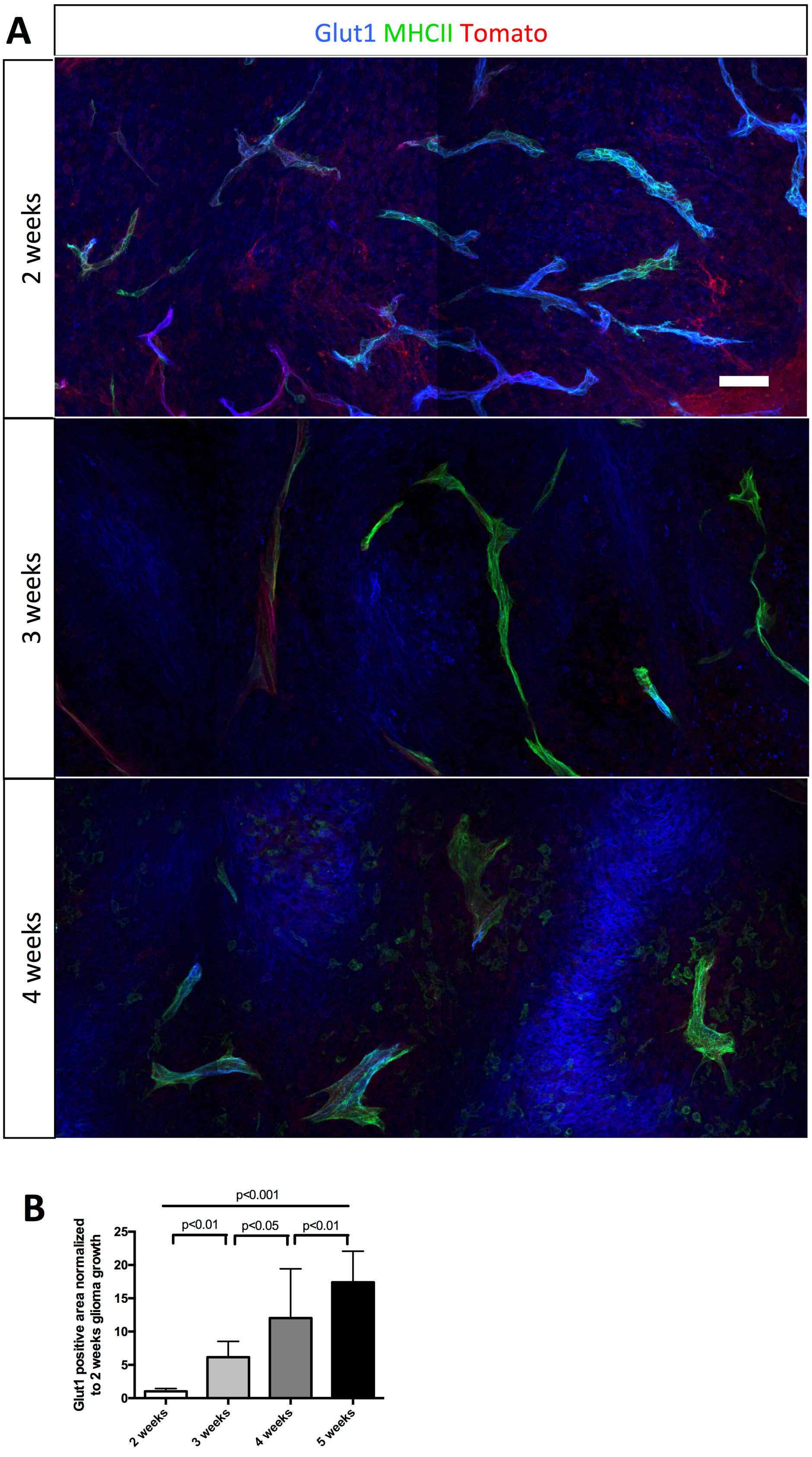
A
Glut1 MHCII Tomato
2 weeks
3 weeks
4 weeks
B
p<0.001
p<0.01
p<0.05
p<0.01
Glut1 positive area normalized to 2 weeks glioma growth
0
5
10
15
20
25
2 weeks
3 weeks
4 weeks
5 weeks

Appendix Figure S8: Hypoxic area increases during glioma growth. A. Glut1 immunohistochemistry on 1, 3 and 4 weeks growth glioma section implanted in ROSA^{mTmG}*::Pdgfb-iCre* mouse. Glut1 positive hypoxic regions extend during glioma progression. Note that 2 weeks glioma growth vessels are still largely covered by Glut1 labeling indicating the functionality of the blood brain barrier (50µm depth stack). **B.** Glut1 hypoxic area quantification during glioma growth (n=5 mice per group). Statistical analysis: B. one-way ANOVA followed by multiple comparisons Tukey's test. Error bars: mean±SD. Scale bar: 200µm.

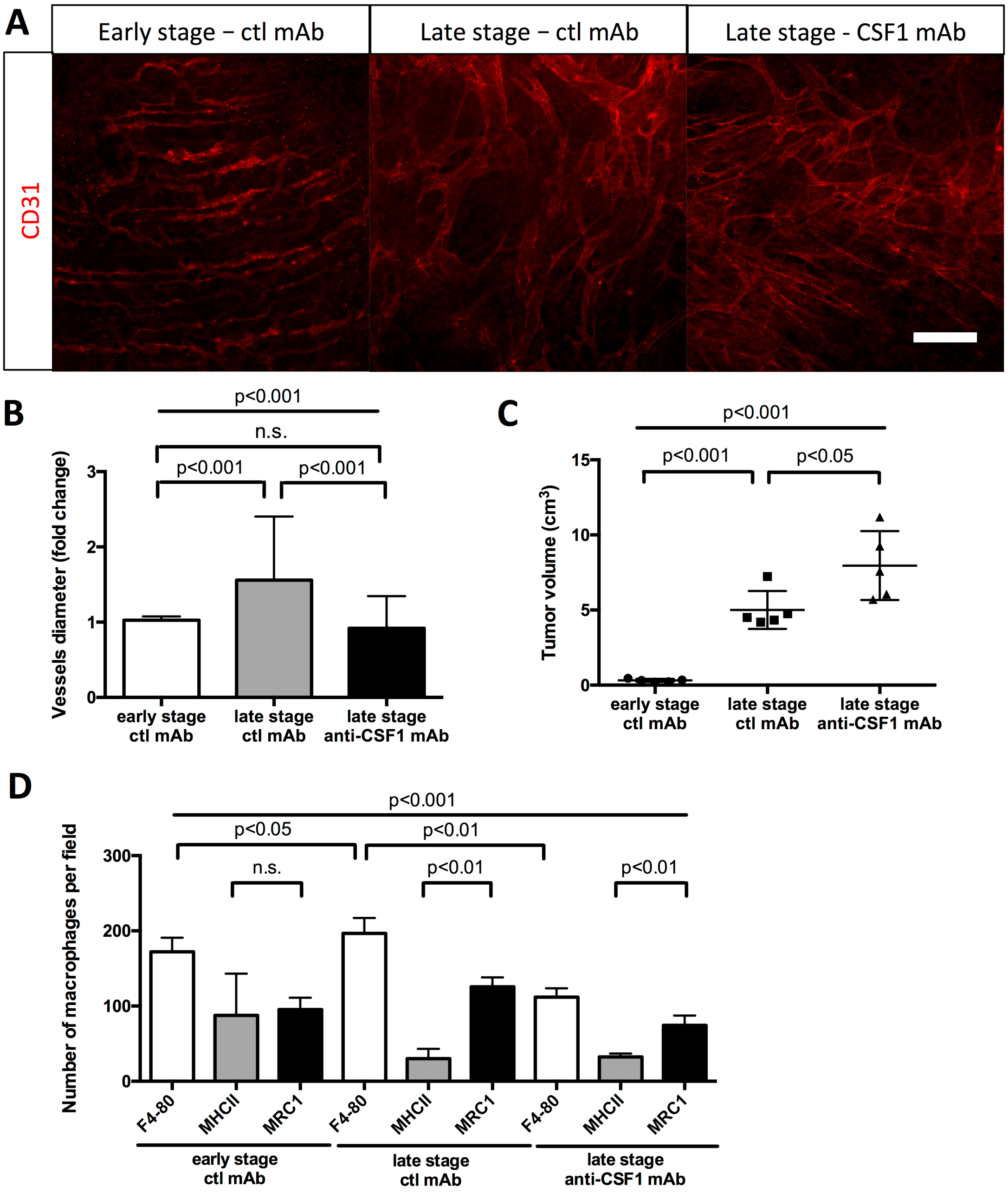

Appendix Figure S9: anti-CSF1 treatment reduce vessel caliber and slightly increase tumor growth in melanoma model. A. CD31 immunohistochemistry on sections of early (4 days) and late (12days) melanomas treated with anti-CSF1 mAb or control mAb. **B.** Blood vessels diameter quantification in melanoma tumor model in response to anti-CSF1

mAb treatment. Anti-CSF1 antibody treatment induces a 1.7 fold decrease in vessel caliber compared to control antibody (n=5 mice per group) (50μm depth stack). **C.** Tumor volume analysis over tumor progression in response to anti-CSF1 mAb. **D.** F4/80, MHCII and MRC1 immunohistochemistry on early and late stage growth melanoma sections implanted in wild-type bl6 mouse and in response to anti-CSF1 mAb. M1 macrophages are as abundant as M2 macrophages at early stage and their number decreases at late stage. M2 macrophages accumulate over tumor progression. Anti-CSF1 mAb treatment decrease macrophages population in melanoma tumor model, but doesn't seems to orientate macrophages polarity. Statistical analysis: B.C.D. one-way ANOVA followed by multiple comparisons Tukey's test. Error bars: mean±SD. Scale bar: 70μm.
