# Supplementary material for: Dynamic stroma reorganization drives blood vessel dysmorphia during glioma growth
Source: EMBO Mol Med. 2017 Oct 16;9(12):1629–45. doi: 10.15252/emmm.201607445 (PMC5709745; doi:10.15252/emmm.201607445)
Supplement: Supplementary file 2 — Expanded View Figures PDF [file EMMM-9-1629-s002.pdf]

Expanded View Figures

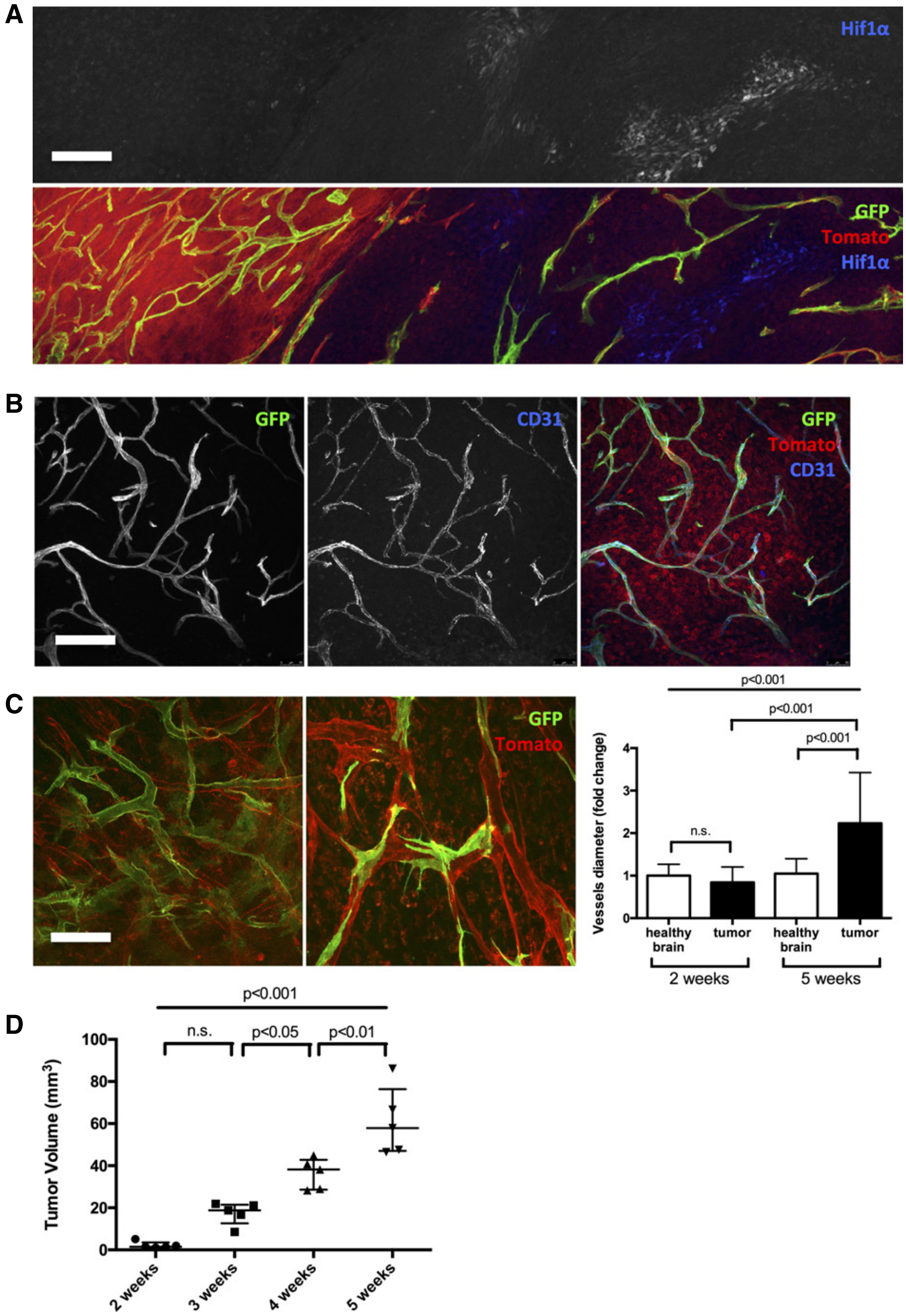

Figure EV1. Blood vessel dysmorphia arises during glioma growth.

A HIF1α immunohistochemistry on 5-week growth glioma in ROSA^{mTmG}::*Pdgfb-iCre* mouse (50-μm depth stack).

B CD31 immunohistochemistry on 5-week growth glioma in ROSA^{mTmG}::*Pdgfb-iCre* mouse revealing the endothelial cell specificity of the *Pdgfb-iCre* induced recombination (50-μm depth stack).

C Representative images of two-photon live imaging on 2- and 5-week growth glioma implanted in ROSA$^{mT/mG}$::*Cdh5-iCre* mouse (350-μm depth stack). Note differences in network complexity and the quantified vessel diameter increase (n = 6 mice per group).

D Tumor volume quantification during glioma growth (n = 5 mice per group).

Data information: Statistical analysis: (C, D) one-way ANOVA followed by multiple comparisons Tukey's test. Error bars: (C) mean ± SD; (D) median interquartile. Scale bars: (A) 250 μm; (B, C) 150 μm.

© 2017 The Authors

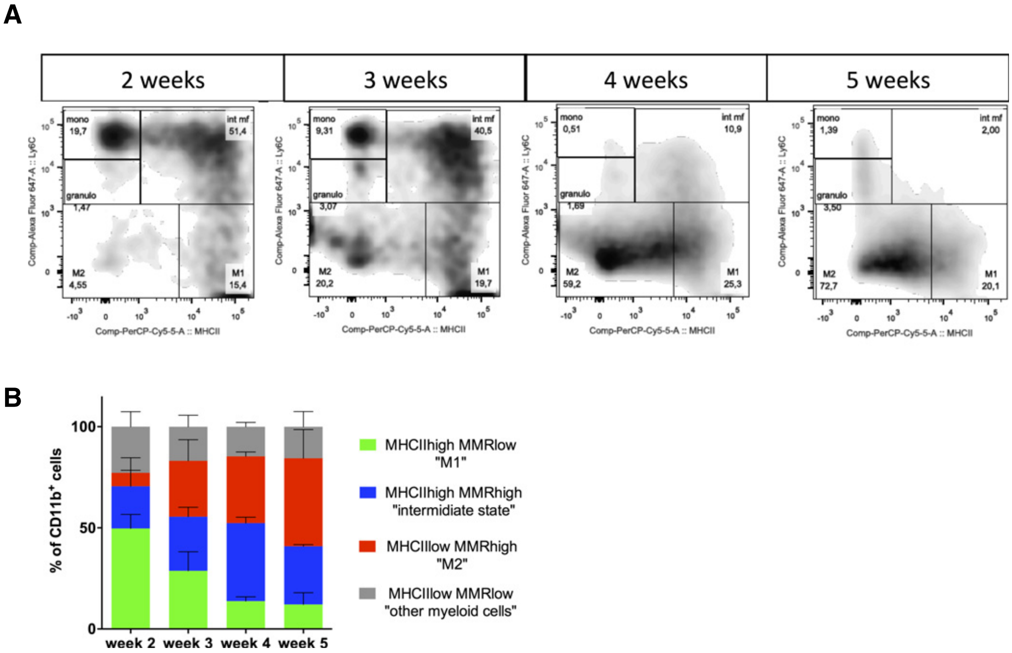

Figure EV2. Macrophage polarization FACs analysis.

A Identification of distinct myeloid subsets in single-cell suspensions of 2-, 3-, 4-, and 5-week glioma (subsets in gated $CD11b^+$ cells).

B Flow cytometry analysis confirming a switch in macrophages polarization over glioma growth (n = 3 mice per group). Error bars: mean ± SD.

 © 2017 The Authors

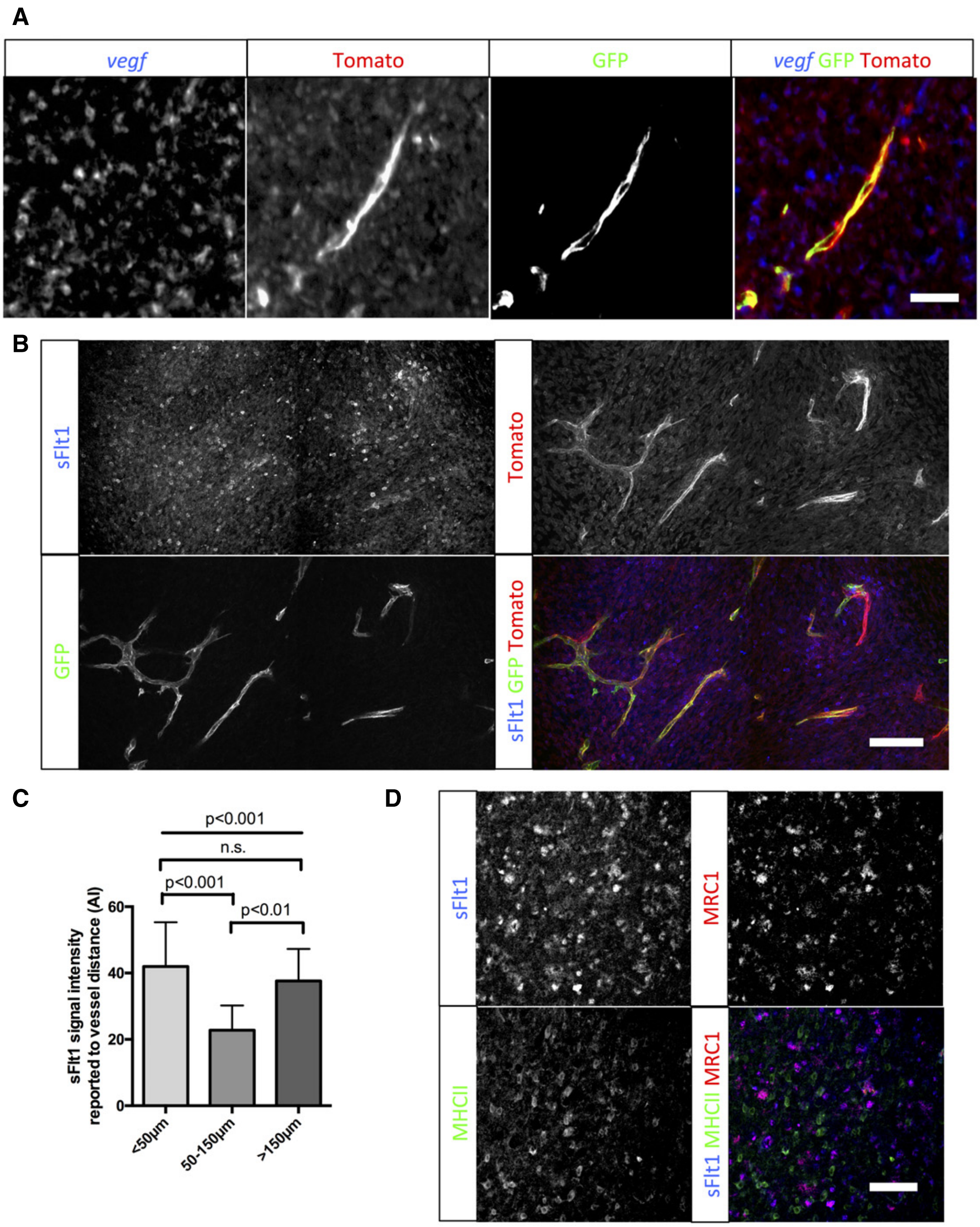

Figure EV3. Late-stage glioma M2 macrophages express and present VEGF close to blood vessels.

A *vegf in situ* hybridization on sections of 5-week growth glioma in ROSA^{mTmG}::*Pdgfb-iCre* mouse. Tomato-positive macrophages surrounding blood vessels are positive for the *vegf*-specific RNA probe (5-µm depth stack).

B sFlt1 binding assay on section of 5-week growth glioma in ROSA^{mTmG}::*Pdgfb-iCre* mouse. At late-stage tumor growth, M2 macrophages surrounding blood vessels present very high amounts of VEGF (binding sFlt1) to the neighboring endothelial cells (50-µm depth stack).

C Quantification of sFlt1 binding accordingly to vessel distance (n = 5 mice).

D sFlt1 binding assay together with MHCII and MRC1 immunohistochemistry on section of 3-week growth glioma in wild-type mice. sFlt1 binds to MRC1-positive macrophages, but not MHCII-positive macrophages (n = 5 mice per group).

Data information: Statistical analysis: (C) one-way ANOVA followed by multiple comparisons Tukey's test. Error bars: mean ± SD. Scale bars: (A) 50 µm; (B) 250 µm; (D) 150 µm.

© 2017 The Authors

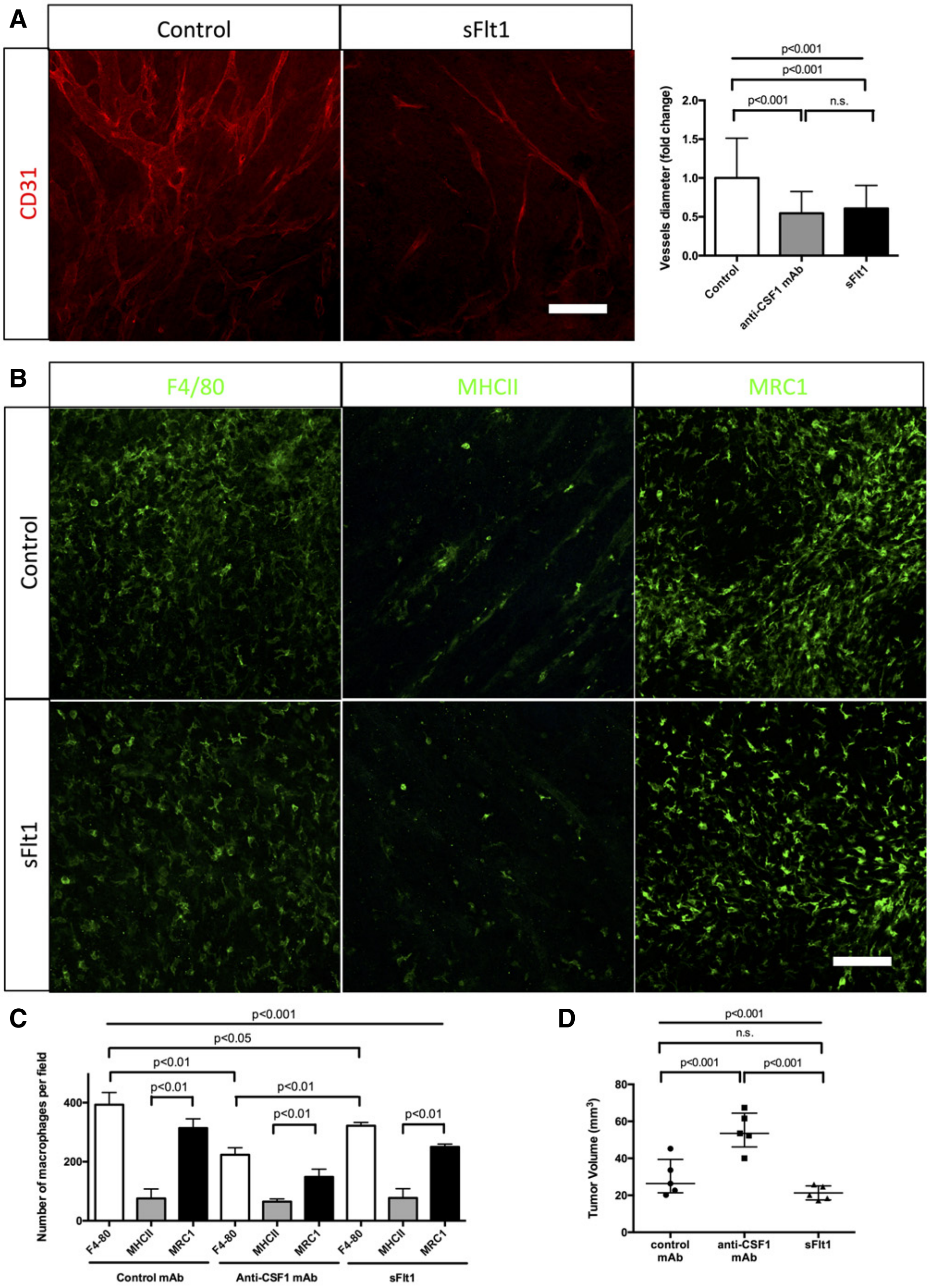

Figure EV4. VEGF trapping with sFlt1 treatment normalizes the vasculature and reduces macrophages recruitment without significantly affecting tumor growth.

A CD31 immunohistochemistry on sections of 4-week growth glioma from mice treated with sFlt1. As for anti-CSF1 antibody treatment, sFlt1 treatment induces a twofold decrease in vessel caliber compared to control antibody (n = 5 mice per group) (50-μm depth stack). Blood vessels significantly dilate over tumor growth.

B F4/80, MHCII, and MRC1 immunohistochemistry on early- and late-stage growth melanoma sections implanted in wild-type bl6 mouse. M1 macrophages are as abundant as M2 macrophages at early stage and their number decreases at late stage. M2 macrophages accumulate over tumor progression

C Macrophages quantification in response to sFlt1 and anti-CSF1 mAb treatments. anti-CSF1 mAb and sFlt1 treatments decrease macrophages population in melanoma tumor model, but do not seem to affect macrophages polarity (n = 5 mice per group).

D Tumor volume analysis in response to sFlt1 and anti-CSF1 mAb treatments (n = 5 mice per group).

Data information: Statistical analysis: (A, C, D) one-way ANOVA followed by multiple comparisons Tukey's test. Error bars: (A, C): mean ± SD; (D) median interquartile. Scale bars: (A, B) 70 μm.

 © 2017 The Authors

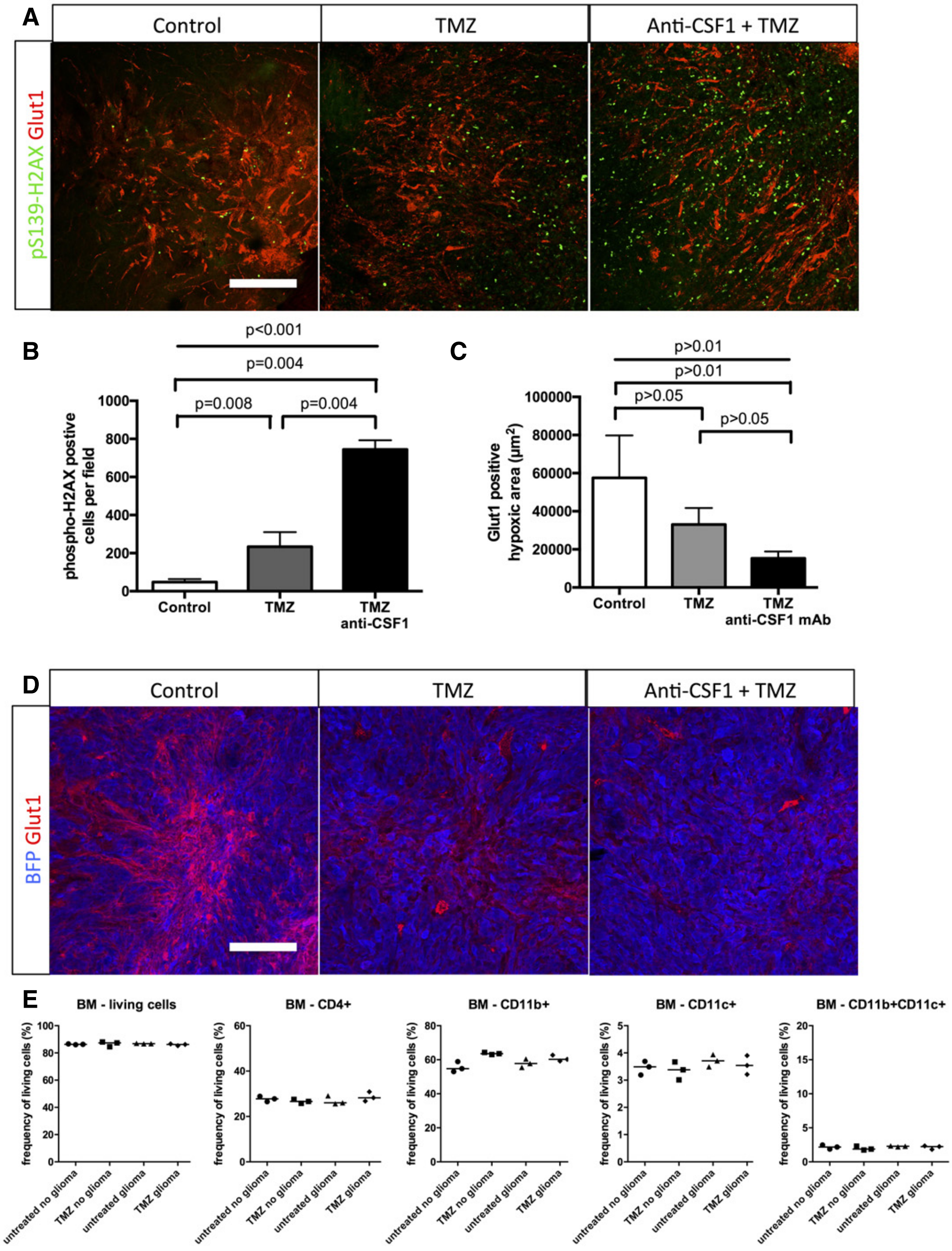

Figure EV5. Combining macrophage depletion with chemotherapy increases DNA damage.

A,B Phospho-H2AX and Glut1 immunohistochemistry on 3-week growth glioma in wild-type mice treated with anti-CSF1 antibody in combination with temozolomide chemotherapeutic agent induces a higher tumor cell death efficiency with (B) a significant increase in DNA damage-positive tumor cell labeled with anti-phospho-H2AX (50-µm depth stack) (n = 6 mice per group).

C Quantification of (D). (n = 6 mice per group).

D Glut1 immunohistochemistry on 3-week growth glioma in wild-type mice treated with anti-CSF1 antibody in combination with temozolomide chemotherapeutic agent showing a significant decrease in hypoxic area in response to temozolomide which is potentiated by anti-CSF1 Ab treatment

E Immune cells population FACs analysis in bone marrow from glioma-bearing mice treated or not with temozolomide (n = 3 mice per group).

Data information: Statistical analysis: (B, C) one-way ANOVA followed by multiple comparisons Tukey's test. Error bars: (B, C) mean ± SD; (E) median. Scale bars: (A, D) 100 µm.
