# Supplementary material for: Dynamic stroma reorganization drives blood vessel dysmorphia during glioma growth
Source: EMBO Mol Med. 2017 Oct 16;9(12):1629–45. doi: 10.15252/emmm.201607445 (PMC5709745; doi:10.15252/emmm.201607445)
Supplement: Supplementary file 3 — Table EV1 [file EMMM-9-1629-s003.docx]

**Table EV1: Patients description.**

| Patient  nb | Pathology | WHO  grade | Age at  surgery |
| --- | --- | --- | --- |
| 1  2  5  6  8  9  10  11  14  15  16  19  20  21  22  23  26  30  31  32  33  34  35  36  37  38  39  40  41 | Primary glioblastoma  Primary diffuse oligodendroglioma  Primary oligodendroglioma  Relapsed glioblastoma  Primary glioblastoma  Relapsed glioblastoma  Primary oligodendroglioma  Primary oligodendroglioma  Relapsed glioblastoma  Primary glioblastoma  Primary glioblastoma  Primary oligodendroglioma  Primary glioblastoma  Relapsed glioblastoma  Relapsed glioblastoma  Primary anaplastic astrocytoma  Relapsed glioblastoma  Primary glioblastoma  Relapsed glioblastoma  Primary anaplastic astrocytoma  Primary glioblastoma  Primary glioblastoma  Primary anaplastic astrocytoma  Primary oligodendroglioma  Primary glioblastoma  Primary anaplastic astrocytoma  Primary glioblastoma  Relapsed glioblastoma  Primary glioblastoma | Grade IV  Grade III  Grade II  Grade IV  Grade IV  Grade IV  Grade II  Grade II  Grade IV  Grade IV  Grade IV  Grade II  Grade IV  Grade IV  Grade IV  Grade III  Grade IV  Grade IV  Grade IV  Grade III  Grade IV  Grade IV  Grade III  Grade II  Grade IV  Grade III  Grade IV  Grade IV  Grade IV | 64  56  44  48  68  70  33  39  44  44  64  30  69  63  30  29  64  58  41  32  54  53  70  25  54  31  72  71  79 |
