# Supplementary material for: Dynamic stroma reorganization drives blood vessel dysmorphia during glioma growth
Source: EMBO Mol Med. 2017 Oct 16;9(12):1629–45. doi: 10.15252/emmm.201607445 (PMC5709745; doi:10.15252/emmm.201607445)
Supplement: Supplementary file 4 — Movie EV1 [file EMMM-9-1629-s004.zip › MovieEV1_legend.docx]

**MovieEV1: Tip cell sprouting in early growth glioma.** 2 hours two-photon live imaging on 2 weeks growth glioma implanted in ROSA^mTmG^*::Pdgfb-iCre* mouse. Tip cells (white arrows) extend filopodias.
