# Supplementary material for: Dynamic stroma reorganization drives blood vessel dysmorphia during glioma growth
Source: EMBO Mol Med. 2017 Oct 16;9(12):1629–45. doi: 10.15252/emmm.201607445 (PMC5709745; doi:10.15252/emmm.201607445)
Supplement: Supplementary file 5 — Movie EV2 [file EMMM-9-1629-s005.zip › MovieEV2_legend.docx]

**MovieEV2: Macrophage recruitment in early growth glioma.** 6 hours two-photon live imaging on 2 weeks growth glioma implanted in ROSA^mTmG^*::Csf1r-Mer-iCre-Mer* mouse. Macrophages start accumulating in early growth tumor exhibiting thin caliber blood vessels.
