# Supplementary material for: Dynamic stroma reorganization drives blood vessel dysmorphia during glioma growth
Source: EMBO Mol Med. 2017 Oct 16;9(12):1629–45. doi: 10.15252/emmm.201607445 (PMC5709745; doi:10.15252/emmm.201607445)
Supplement: Supplementary file 7 — Movie EV4 [file EMMM-9-1629-s007.zip › MovieEV4_legend.docx]

**MovieEV4: Endothelial cell synchronized sprouting provokes blood vessel expansion.** 2 hours two-photon live imaging on 5 weeks growth glioma implanted in ROSA^mTmG^*::Pdgfb-iCre* mouse. All endothelial cells extend filopodias in a synchronized manner (white arrows).
